# Rate, timing and predictors of relapse in patients with anorexia nervosa following a relapse prevention program: a cohort study

**DOI:** 10.1186/s12888-016-1019-y

**Published:** 2016-09-08

**Authors:** Tamara Berends, Berno van Meijel, Willem Nugteren, Mathijs Deen, Unna N. Danner, Hans W. Hoek, Annemarie A. van Elburg

**Affiliations:** 1 Altrecht Eating Disorders Rintveld, Wenshoek 4, 3705 WJ Zeist, The Netherlands; 2 Department of Health, Sports & Welfare, Cluster Nursing, Inholland University of Applied Sciences, Research Group Mental Health Nursing, Amsterdam, The Netherlands; 3 Department of Psychiatry, VU University Medical Center, Amsterdam, The Netherlands; 4 Parnassia Psychiatric Institute, Parnassia Academy, The Hague, The Netherlands; 5 Parnassia Psychiatric Institute, Clinical Centre for Acute Psychiatry, the Hague, The Netherlands; 6 Institute of Psychology, Methodology and Statistics Unit, Leiden University, Leiden, The Netherlands; 7 Utrecht Research Group Eating Disorders, Utrecht, The Netherlands; 8 Department of Psychiatry, University Medical Center Groningen, University of Groningen, Groningen, The Netherlands; 9 Department of Epidemiology, Columbia University, Mailman School of Public Health, New York, USA; 10 Department of Social Sciences, Utrecht University, Utrecht, The Netherlands

**Keywords:** Anorexia nervosa, Relapse, Relapse intervention, Relapse prevention, Survival analysis

## Abstract

**Background:**

Relapse is common among recovered anorexia nervosa (AN) patients. Studies on relapse prevention with an average follow-up period of 18 months found relapse rates between 35 and 41 %. In leading guidelines there is general consensus that relapse prevention in patients treated for AN is a matter of essence. However, lack of methodological support hinders the practical implementation of relapse prevention strategies in clinical practice. For this reason we developed the Guideline Relapse Prevention Anorexia Nervosa. In this study we examine the rate, timing and predictors of relapse when using this guideline.

**Method:**

Cohort study with 83 AN patients who were enrolled in a relapse prevention program for anorexia nervosa with 18 months follow-up. Data were analyzed using Kaplan-Meijer survival analyses and Cox regression.

**Results:**

Eleven percent of the participants experienced a full relapse, 19 % a partial relapse, 70 % did not relapse. Survival analyses indicated that in the first four months of the program no full relapses occurred. The highest risk of full relapse was between months 4 and 16. None of the variables remained a significant predictor of relapse in the multivariate Cox regression analysis.

**Conclusion:**

The guideline offers structured procedures for relapse prevention. In this study the relapse rates were relatively low compared to relapse rates in previous studies. We recommend that all patients with AN set up a personalized relapse prevention plan at the end of their treatment and be monitored at least 18 months after discharge. It may significantly contribute to the reduction of relapse rates.

## Background

Anorexia nervosa (AN) is a severe mental disorder with a life-time prevalence among women of 2 % [1, 2] and high mortality rates of 5 % per decade [2, 3]. Relapse is common among AN patients who previously showed full remission of the eating disorder. Studies have reported a wide range of estimates of relapse rates in AN, depending upon the definitions of relapse used, the length of follow-up, and the methodologies employed [4].

Relapse rates in studies with longer follow-up periods, and without targeted prevention strategies differ from 6 to 57 % [5–11].

In three comparable studies on relapse prevention with an average follow-up period of 18 months, full relapse rates of 35, 41 and 41 % were found [4, 12, 13]. These studies demonstrated with survival analyses that the highest risk of relapse was between 4 and 17 months post-treatment. In the Netherlands, where also the present study has been conducted, research by Van Elburg [14] showed a relapse rate of more than 50 % over a period of five years. In none of these studies a structured relapse prevention program was applied.

In leading guidelines in the field of eating disorders [15–17], general consensus exists that relapse prevention in patients with AN is essential. However, a major problem is the lack of structured methods for relapse prevention to support professionals in clinical practice. Therefor the Guideline Relapse Prevention Anorexia Nervosa (GRP) was developed, intended for use by both professionals and patients to apply relapse prevention strategies in a structured manner [18]. This Guideline was implemented in specialized treatment setting for eating disorders in The Netherlands.

The aim of the present study is to examine the rate, timing and predictors of relapse of patients who were treated with the GRP.

## Method

### Design

Cohort study of patients successfully treated for AN included in a relapse prevention program for AN, with a follow-up of 18 months.

### Participants and setting

The following inclusion criteria were applied: in- and outpatients, age 12 years and older, meeting the diagnostic criteria of the DSM-IV [19] for AN or EDNOS clinically referred to as AN (for example women meeting all criteria for AN, except that the individual has menses. In 33 cases the diagnosis was determined according to the DSM-IV criteria and ascertained by eating disorder experts (all psychiatrists), supported by questions from the EDE (Eating Disorder Examination) [20, 21]. In 50 cases the actual EDE interview was administered to confirm the eating disorder diagnosis that was determined by the psychiatrist in accordance with the DSM-IV criteria.

Participants had successfully completed their treatment, were weight restored with a normal (SD) BMI based on their age and height. For inclusion it was further required that they had drawn up a relapse prevention plan (RPP) at the end of their treatment.

Ninety-six patients were eligible to participate in the after-care program between 2009 and 2012, where the Guideline Relapse Prevention Anorexia Nervosa (GRP) was implemented. Thirteen participants did not meet the inclusion criteria: seven participants did not have a complete RPP, two patients were re-admitted for treatment during the drafting of the RPP, two participants refused to participate in the after-care program after making a RPP, one patient moved abroad before starting the after-care program, and one patient was admitted to a closed ward during this study due to severe comorbidity (severe depression with a risk of suicide). The remaining 83 participants were included in the analyses.

The study was carried out in a specialized treatment center for eating disorders in the Netherlands, Altrecht Eating Disorders Rintveld. The treatment provided in this specialized setting is based on the state-of-the-art evidence- and practice-based knowledge as described in three guidelines: The Dutch Multidisciplinary Guideline Eating Disorders [15], the NICE guidelines Eating Disorders [16] and the American Psychiatric Association Practice Guideline: Treatment of Patients with Eating Disorders [17]. Treatment focuses on three areas: (1) eating habits, body weight, and body image; (2) psychological aspects of functioning, such as self-esteem, perfectionism, and traumas; and (3) social functioning within the family system and in society. In our center, patients are basically treated on an outpatient basis, and only admitted for short periods at a time, followed by outpatient treatment. All patients who started the relapse prevention program received prior outpatient treatment. Only when remission was reached during outpatient treatment were patients eligible for participation in the aftercare program. Comorbidity is managed either in the center itself or by co-treatment in a different specialized center.

### Definition of relapse

In the present study the primary outcome was the occurrence of relapse. The distinction was made between full and partial relapse. A full relapse was defined as: BMI <18.5 for adults and SD BMI < -1 for adolescents, together with full recurrence of the core diagnostic symptoms of AN according to DSM-IV criteria, in the first instance assessed by the professional, and next confirmed in a multidisciplinary consensus meeting. In case of confirmation, this formed an indication for renewed treatment. Partial relapse was defined as the re-occurrence of one or more core diagnostic symptoms of AN, after a previous positive response to treatment. As a response to the re-occurrence of symptoms, a temporary intensification of the after-care program for a period up to three months was needed to achieve full recovery again. If a longer intensification of the program was needed the relapse was classified as a full relapse.

### The guideline relapse prevention anorexia nervosa (GRP)

The primary aim of the guideline is that the professional, patient and her relatives work closely together to gain a better understanding of the patient’s individual process of relapse. Triggers and early warning signs that preceded previous relapses are identified and elaborated for the individual patient, and actions are formulated that can be performed in the event of a new impending relapse. All this information is summarized in a Relapse Prevention Plan (RPP). The essence of the relapse prevention strategy is to ensure that appropriate action is taken as early as possible when early warning signs of relapse occur.

The guideline is made up of three parts: (a) a theoretic framework for relapse and relapse prevention, developed on the basis of both the literature and practical experience of experts and patients, leading to conclusions and recommendations for clinical practice; (b) a practical manual for the professional; and (c) a workbook for patients.

For a complete description of the application of the GRP, see the case report by Berends, van Meijel & van Elburg [22]. The GRP is freely accessible via the internet.

Drawing up a fully fledged relapse prevention plan requires approximately six meetings of patient, relatives and the professional. Practical experience with the application of the GRP showed that individual sessions should last approximately 45 min and should preferably be scheduled every other week. After each session, the patient receives homework assignments, to be carried out either individually or together with relatives.

After a RPP is drawn-up, the aftercare-program starts. This is a low-frequent individual program and has a minimum duration of 18 months. During the aftercare-visits the condition of the patient is thoroughly monitored and discussed. Two scenarios can occur during these visits: 1) The patient is stable, in which case the focus is on maintaining this stable condition by promoting good physical health and optimal personal and social functioning. Actual or possible stressful life events in the near future are discussed and anticipated on. 2) The patient shows one or more early signs of impending relapse, in which case the main focus during the visit is on obtaining a thorough understanding of the actual triggers of relapse, and how to deal with these in order to promote recovery. In this context specific arrangements are made and actions are planned, based on the content of the previously established relapse prevention plan (RPP).

The frequency of the aftercare visits depends on the patient’s condition and the need for treatment and care. For example, patients who are stable will come for a visit after four to six months. If the patient is less stable the visits can be planned every two months. The patient and the professional can decide to extend the aftercare period after 18 months in case of prolonged vulnerability to relapse, with a maximum of five years.

The visits last 45 min and are attended by both the patient and her relatives. At each visit the patient is weighed and her condition is evaluated. During the visit, two main topics are discussed, i.e., psychological and social functioning (school, friends, sports, overall moods, etc.) and the presence of AN-symptoms (anorectic cognitions, abnormal eating habits, excessive exercise pattern et cetera). Based on this information, the RPP is updated if necessary. At the end of the visit a new appointment is made for the next visit. The patient’s record contains the following details of each visit: weight, possible stage of relapse, and the arrangements made during the visit.

Between the formal visits, a patient or her relatives can contact the professional at any time in case of need for help.

### Data collection

Data were collected on:

1. Demographic and clinical characteristics: age, age of onset, severity of the eating disorder, treatment duration, duration of the eating disorder, BMI, in- or outpatient treatment, number of sessions in the aftercare program, subtype of AN (restrictive type (ANR) or binge/purging type (ANBP)), and comorbidity (as ascertained by psychiatrists at the start of treatment and confirmed in a consensus meeting by the clinical team).
2. Data concerning full and partial relapse: weight, stage of relapse, and the agreements made during the visit.
  When a participant had a full relapse the indication for renewed treatment was documented.
  When a participant had a partial relapse the stage of relapse was documented, as well as the intensification of the aftercare-visits. When a participant crossed the three-month duration of partial relapse, it was registered as a full relapse.

### Data-analysis

1. Kaplan-Meier survival analysis was used to analyze the rate and timing of full relapse.
2. Demographic and clinical characteristics between the group of patients with either full or partial relapse, and the group of patients with no relapse are presented with percentages and means.
3. In order to identify significant predictors of relapse, Cox regression was used to assess the predictive value of demographic and clinical characteristics with respect to relapse. First, the variables were tested univariate, after which predictors with a *p-value* < .10 were entered in a multivariate Cox regression model. SPSS for Windows (version 21.0) was used to perform all statistical procedures.

## Results

### Baseline characteristics participants

The 83 participants had a mean age of 17.9 years (*SD* = 4.45), measured at the start of the aftercare-program. Their mean BMI was 16.4 kg/m^2^ (*SD* = 2.13) at the start of the initial treatment, and 20.0 kg/m^2^ (*SD* = 1.54) at the end of treatment, which is at the start of the aftercare-program. Since 58 participants were younger than 19 years, SD BMI was collected and therefor converted to BMI. The mean age of onset was 14.3 years (*SD* = 3.40). Of the participants 84.3 % (*n* = 70) was diagnosed with anorexia nervosa restrictive type (ANR), and 15.7 % (*n* = 13) with anorexia nervosa binge purging type (ANBP). According to the DSM-5 severity scale, 13.3 % of the participants had a mild disorder at the start of treatment, 18.1 % moderate, 12 % severe and 56.6 % extreme. There were no significant differences between full and partial relapsers concerning severity of the disorder. The average time of participation in the aftercare-program was 18.4 months (*SD* = 4.39). The mean number of sessions during the aftercare program for the non-relapse group was 4.14 (*SD* = 1.89) sessions; for the partial relapse group it was significantly higher at 6.69 (*SD* = 5.00) sessions (*p* = 0.008). For the full relapse group, the mean number of sessions was 7.11 (*SD* = 7.49) sessions (*p* = 0.269).

### Rate and timing of relapse

During the aftercare program, 10.8 % (*n* = 9) of the participants experienced a full relapse, whereas 19.3 % (*n* = 16) had a partial relapse and 69.9 % (*n* = 58) did not relapse.

**Fig. 1:**
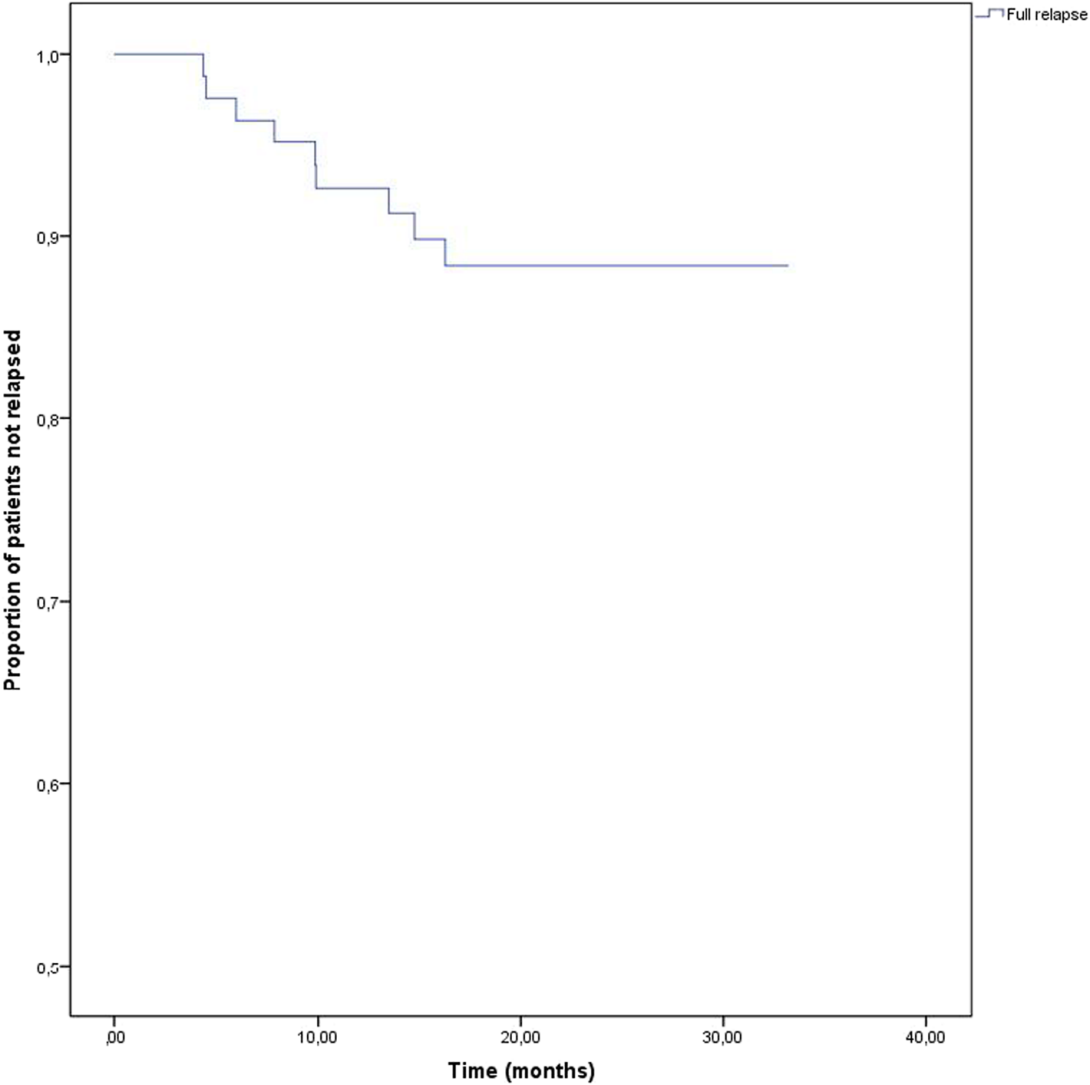
Survival function for full relapse

Figure 1 presents the survival curve for the 83 participants showing full relapse. No full relapses occurred in the first four months of the program. The highest risk of full relapse was between months 4 and 16. After 16 months no full relapse occurred while 61 participants still participated in the aftercare-program at that point in time.

### Identification of predictors of relapse

In order to identify significant predictors of relapse, first univariable analyses were performed on demographic and clinical characteristics (Tables 1 and 2). ‘Duration of treatment’ (*p* = 0.007), ‘Type of treatment’ (*p* = 0.039) and ‘Age’ (*p* = 0.034) were the only variables to significantly predict time until relapse. When entered in a multivariate Cox regression model none of the variables were statistically significant.

**Table 1:** Demographic and clinical characteristics of the participants per group (full and partial relapse vs. non-relapse) including outcome univariate Cox regression

Variable	Full and partial relapse group (*n* = 25)	Non relapse group (*n* = 58)	Univariate Cox regression *p* value	Univariate Cox regression Exp(B)
Onset eating disorder (years)	15.1	14.1	0.440	1.034
Duration of the eating disorder (years)	3.7	3.4	0.447	1.055
Duration of treatment before the aftercare-program (months)	25.6	16.2	0.007*	1.028
BMI at start treatment (kg/m^2^)	16.3	16.4	0.621	0.954
BMI at start of aftercare program	19.9	20.0	0.776	0.965
BMI at the end of aftercare program	19.9	20.4	0.166	0.865
Age 18 years or younger	22,4 % (*n* = 13)	77,6 % (*n* = 45)	/	/
Age 19 years or older	48 % (*n* = 12)	52 % (*n* = 13)	0.034*	2.344
Subtype ANBP	23.1 % (*n* = 3)	76.9 % (*n* = 10)	/	/
Subtype ANR	31.4 % (*n* = 22)	68.6 % (*n* = 48)	0.709	1.259
Type of treatment before the aftercare-program (patients received only outpatient treatment)	32 % (*n* = 8)	56,9 % (*n* = 33)	/	/
Type of treatment before the aftercare-program (patients received in- and outpatient treatment)	68 % (*n* = 17)	43.1 % (*n* = 25)	0.039*	2.425
Severity eating disorder DSM 5: Mild	16 % (*n* = 4)	12.1 % (*n* = 7)	0.764	1.179
Severity eating disorder DSM 5: Moderate	12 % (*n* = 3)	20.7 % (*n* = 12)	0.321	0.543
Severity eating disorder DSM 5: Severe	8 % (*n* = 2)	13.8 % (*n* = 8)	0.447	0.571
Severity eating disorder DSM 5: Extreme	64 % (*n* = 16)	53.4 % (*n* = 31)	0.266	1.591
Comorbidity	52 % (*n* = 13)	51.7 % (*n* = 30)	0.948	1.027
Anxiety disorder	0 % (*n* = 0)	6.9 % (*n* = 4)	0.458	0.046
Depressive disorder	24 % (*n* = 6)	8.6 % (*n* = 5)	0.151	1.963
Dysthymia	0 % (*n* = 0)	5.2 % (*n* = 3)	0.515	0.047
Obsessive compulsive disorder	0 % (*n* = 0)	6.9 % (*n* = 4)	0.426	0.046
Parent-child Relational Problem	24 %(*n* = 6)	19 % (*n* = 11)	0.363	1.533
Autism	0 % (*n* = 0)	1.7 % (*n* = 1)	0.660	0.048
Personality disorder	8 % (*n* = 2)	10.3 % (*n* = 6)	0.730	0.775

*BMI* body mass index, *ANR* anorexia nervosa restrictive, *ANBP* anorexia nervosa binge purge

﻿*statistically significant

**Table 2:** BMI of the participants with a full relapse compared to the non full relapse group, including test outcome/statistics

Variable	Full relapsed group (*n* = 9)	Non full relapsed group (*n* = 74)	T-test *p*-value	Fisher’s exact test (2-sided) *p*-value
BMI at start aftercare-program	19.46	20.07	0.12	–
BMI at the end of the aftercare-program	18.47	20.52	0.002*	–

*statistically significant

## Discussion

The purpose of this study was to examine the rate, timing and predictors of relapse in a group of recovered AN patients who participated in an aftercare-program using the Guideline Relapse Prevention Anorexia Nervosa. The full relapse rate for AN was 11 %, which is much lower than the relapse rates found in previous studies with an average follow-up period of 18 months, showing full relapse rates of 35 % [4], 41 % [12] and 41 % [13]. In these studies no structured methods for relapse prevention were applied.

Of the participants in the present study who experienced the first signs of a relapse (30 %), 19 % recovered within a three-month period and only 11 % relapsed fully. When experiencing the first signs of relapse as described in their RPP, these patients contacted their professional within a week. It was therefore possible to intervene at an early stage, guided by the predefined actions elaborated in the RPP.

Although no definitive conclusions about the effectiveness of the intervention can be drawn in this cohort study, the findings support our hypothesis that working with the guideline relapse prevention has a preventive effect on the occurrence of full relapse in patients with AN. When combining the rates of both partial and full relapse, the percentage of 30 % is in the lower range of relapse rates found in other studies.

The survival analysis shows the timing of full relapses in this sample and indicates that within the first four months after discharge (and after starting with the RPP) no full relapses occurred, whilst the highest risk of full relapse was between 4 and 16 months after discharge. When combining the data concerning timing of partial and full relapse, the findings indicate that the risk of relapse is increased throughout the entire period of 18 months, suggesting that monitoring of early signs of relapse is necessary during this complete period.

For the identification of predictors of relapse, the univariate Cox regression between the two groups (the partial and full relapse group versus the non-relapse group) on demographic and clinical variables revealed that they differed significantly on three variables. The first variable was ‘Duration of treatment before the start of the aftercare program’. The longer a patient was in treatment, the higher the risk of relapse. Duration of treatment could point to relatively high vulnerability of patients and severity of illness, thus leading to a higher risk of relapse. This subgroup of patients should be offered extra attention during aftercare, with proper information about the increased risk of relapse, and intensive monitoring for a period of at least 18 months. The second variable was ‘Type of treatment before the aftercare program’, either outpatient treatment only or a combination of in- and outpatient treatment. Patients who received both in- and outpatient treatment had a higher risk of relapse than patients who received outpatient treatment only. It can be assumed that patients who require both inpatient and outpatient treatment are more severely affected by the eating disorder, compared to patients who require outpatient treatment only, leading to a higher risk of relapse. These findings are consistent with other studies on relapse [6, 11, 12, 23]. The third variable was’Age’. Patients older than 19 years had a higher risk of relapse, based on the univariate regression analysis. Previous studies also show higher relapse rates within adults [4, 7–9, 11, 12]. In the end none of the variables remained a significant predictor of relapse in the multivariate Cox regression analysis. The absence of unique contribution of any of the predictors might be due to correlation between these predictors.

A limitation of this retrospective study is that no standardized diagnostic instrument was used to determine relapse or detect predictors of relapse. The determination of relapse was based on BMI and the core diagnostic symptoms of AN according to the DSM IV, assessed by the clinical expert on eating disorders and next confirmed in a multidisciplinary consensus meeting. We do recommend the use of a standardized diagnostic interview in future prospective research to assess the occurrence of relapse. The characteristics and variables used in our analyses were collected from the participants’ files. For this reason we could not explore the predictive value for relapse of relevant variables. Future prospective research on predictors of relapse should include validated questionnaires to systematically examine these variables are possible predictors of relapse.

These findings have clinical implications for relapse prevention of AN. The general guidelines for the treatment of AN lack methodological support in the practical implementation of relapse prevention strategies in clinical practice. From the present study, there are indications that the GRP provides an effective tool for relapse prevention. Our tentative conclusion is that when early recognition strategies are applied in case of impending relapse, followed by targeted interventions to prevent further deterioration, the risk of a full relapse will decrease. This increases the chance for patients to eventually reach a stable and lasting recovery. The low relapse rate in our study supports the effectiveness of this strategy, although a limitation of this study is the non-experimental study design.

It is recommended to educate patients that the first 18 months after discharge is a high-risk period for relapse, requiring continuous efforts to prevent relapse using early recognition and intervention techniques. Motivational techniques applied by professionals are needed, in order for the patients to maintain awareness of the increased risk of relapse, which with proper preventive activities can be managed for a significant proportion of cases.

The strength of this study is the relatively large sample size of 83 participants. The research on relapse is scarce and sample sizes are usually small. A limitation of this study is the lack of a control group with randomization of patients. Our study was set up to descriptively obtain insight into relapse rates, timing and predictors. Despite the positive trends we found in this study concerning these relapse rates, the effectiveness of the guideline could therefor not be determined unequivocally. Future research would have to make use of a controlled study design to confirm the effectiveness of the proposed relapse prevention strategy.

## Conclusions

Working with the relapse prevention guideline offers structured support to prevent relapse in patients with AN. We recommend that all patients with AN set up a RPP at the end of their treatment, with regular monitoring for a period of at least 18 months after discharge. Patients gain a better understanding of the relapse process when drawing up an RPP and working with it, enabling them to develop self-management skills to independently influence the course of their illness and ultimately prevent the occurrence of relapse.

## Abbreviations

AN: Anorexia nervosa
ANBP: Anorexia nervosa binge purge
ANR: Anorexia nervosa restrictive
DSM-IV: Diagnostic and statistical manual of mental disorders IV
EDE: Eating disorder examination
EDNOS: Eating disorder not otherwise specified
GRP: Guideline relapse prevention
RPP: Relapse prevention plan
(SD) BMI: (Standard deviation) Body mass index
SPSS: Statistical package for the social sciences
